# Automation of human pluripotent stem cell differentiation toward retinal pigment epithelial cells for large-scale productions

**DOI:** 10.1038/s41598-019-47123-6

**Published:** 2019-07-23

**Authors:** Florian Regent, Lise Morizur, Léa Lesueur, Walter Habeler, Alexandra Plancheron, Karim Ben M’Barek, Christelle Monville

**Affiliations:** 1 INSERM U861, I-Stem, AFM, Institute for Stem cell Therapy and Exploration of Monogenic diseases, 91100 Corbeil-Essonnes, France; 2 UEVE U861, I-Stem, AFM, Institute for Stem cell Therapy and Exploration of Monogenic diseases, 91100 Corbeil-Essonnes, France; 3 CECS, I-Stem, AFM, Institute for Stem cell Therapy and Exploration of Monogenic diseases, 91100 Corbeil-Essonnes, France

**Keywords:** Embryonic stem cells, Stem-cell differentiation, Differentiation

## Abstract

Dysfunction or death of retinal pigment epithelial (RPE) cells is involved in some forms of Retinitis Pigmentosa and in age-related macular degeneration (AMD). Since there is no cure for most patients affected by these diseases, the transplantation of RPE cells derived from human pluripotent stem cells (hPSCs) represents an attractive therapeutic alternative. First attempts to transplant hPSC-RPE cells in AMD and Stargardt patients demonstrated the safety and suggested the potential efficacy of this strategy. However, it also highlighted the need to upscale the production of the cells to be grafted in order to treat the millions of potential patients. Automated cell culture systems are necessary to change the scale of cell production. In the present study, we developed a protocol amenable for automation that combines in a sequential manner Nicotinamide, Activin A and CHIR99021 to direct the differentiation of hPSCs into RPE cells. This novel differentiation protocol associated with the use of cell culture robots open new possibilities for the production of large batches of hPSC-RPE cells while maintaining a high cell purity and functionality. Such methodology of cell culture automation could therefore be applied to various differentiation processes in order to generate the material suitable for cell therapy.

## Introduction

Human pluripotent stem cells (hPSCs), including human embryonic stem cells (hESCs) and human induced pluripotent stem cells (hiPSCs) are characterized by unlimited self-renewal and their ability to differentiate into any cell type. Due to these properties, extensive efforts have been done to use them as a source material for cell therapy to repair damaged tissues. At the forefront of cell therapy, the replacement of retinal pigment epithelium (RPE), the monolayer of pigmented cells localized between the neural retina and the choroid, acts as a proof of concept. RPE cells play crucial roles in sight and their dysfunction or their loss may engender the secondary loss of photoreceptors^1^. RPE cells are altered in 5–6% of Retinitis Pigmentosa cases (RP, a group of rare hereditary diseases) and in Age-related Macular Degeneration (AMD)^2,3^. AMD is the leading cause of blindness in developed countries with more than 150 million people affected worldwide, a figure that will increase in the coming years^4^. It can be classified into two groups, dry (atrophic) or wet (exudative), which is based on the presence of a choroidal neovascularization. While the understanding of the molecular mechanisms underlying wet AMD, which accounts for approximately 10–15% of AMD patients, led to the development of effective anti-VEGF drugs, there is still no treatment for dry AMD and for most of RPs^5,6^. As such, the transplantation of RPE cells derived from human pluripotent stem cells (hPSC-RPE) represents an attractive strategy for treating retinal degenerative diseases^7,8^.

hPSCs spontaneously differentiate into RPE cells after removal of basic fibroblast growth factor (bFGF), used to maintain the pluripotency state, from the culture medium^9–11^. The distinctive cobblestone morphology of RPE cells as well as their pigmentation allow to manually collect pigmented areas that appear upon differentiation of hPSCs to obtain a pure population of hPSC-RPE cells. Such approach of RPE cell production is used as cell replacement material in on going and planned clinical trials^12–16^. However, this spontaneous method remains fastidious, inefficient and time consuming (8 to 12 weeks of hPSCs differentiation) making it incompatible with the industrial large-scale production which is required to treat the potential millions of patients.

During the last ten years, several teams have developed improved differentiation protocols by combining the use of an increasing number of cytokines and small molecules selected on the basis of results obtained from developmental studies^17–20^. One of the quickest and most efficient protocol was published by Leach *et al*. in 2015^20–22^. Following data demonstrating that RPE and neural retina progenitors (NRPs) have the same embryonic origin, they combined a protocol allowing the efficient differentiation of NRPs^23^ with previously described RPE inducing factors such as Nicotinamide (NIC) and Activin A^24^, a member of the TGF-β super family. Using this method, they obtained a large majority of cells expressing the pigmentation marker PMEL17 after 14 days of differentiation allowing bypassing manual enrichment of pigmented cells. It is, however, important to note that even with this protocol the banking of mature RPE cells is only performed after several additional weeks of maturation (Day 84)^22^.

Therefore, although the differentiation of hPSCs into RPE cells became more efficient during the last years, it still remains a long and laborious process requiring meticulous manipulations from hPSCs thawing to hPSC- RPE cell banking. Many cell culture parameters, such as seeding homogeneity, the time spent by the cells out of the incubator or the method used to isolate pigmented clumps, could impact on the proliferation and the differentiation of hPSCs. Thus, manual processing implies operator to operator variability^25,26^ and the quality of hPSCs and the efficiency of their differentiation into RPE cells are currently highly dependent on technical skills. In this regard, automation should not only allow scaling up the production of hPSC-RPE cells but should also increase its robustness. It could enable larger and more reliable cell production for clinical and disease modeling applications. Robots, such as Cell^host^ system or CompacT SelecT system, as well as appropriate programing were already developed for the amplification of hPSCs. Differentiation into mesenchymal and neural stem cells has been also implemented in such systems^27–32^. Until recently, the requirement of a manual enrichment to obtain a pure population of hPSC-RPE cells prevented the use of these automated systems for the differentiation of this cell type. However, the recent development of protocols allowing efficient differentiation of hPSCs into RPE cells offers now the possibility to automate the production of these cells.

Thus, our aim was to develop a fully automated process allowing the large-scale production of hPSC-RPE cells. Considering that, in addition to considerably complicate the process, the use of numerous growth factors and small molecules on a large scale should be very expensive, especially for an automated process which requires significant dead volumes, we implemented a simplified RPE differentiation protocol amenable for automation that only requires the treatment of hPSCs with NIC, Activin A and CHIR99021, an activator of the Wnt canonical pathway, in a sequential manner. Our protocol recapitulates the main steps of retinal development and is sufficient to obtain a pure population of RPE cells without manual enrichment. We then programmed a culture robot to automate this protocol in order to upscale the production process. 16 billion of mature and functional RPE cells could now be produced within 12 weeks with only one round of production. Such efficient and reproducible automated protocol should be useful for the treatment of the millions of patients affected by RPE associated retinal degeneration.

## Results

### Sequential use of nicotinamide, activin A and Chir99021 improves RPE differentiation by recapitulating the main steps of retinal development

In an effort to simplify previous directed differentiation protocols for automation, we evaluated whether the simple use of NIC, Activin A and Chir99021 in a sequential manner (referred as “directed protocol”) improves RPE cell differentiation of adherent hESCs enough to bypass manual enrichment. We compared the efficiency of our “directed protocol” with the one of the classical spontaneous differentiation.

**Figure 1:**
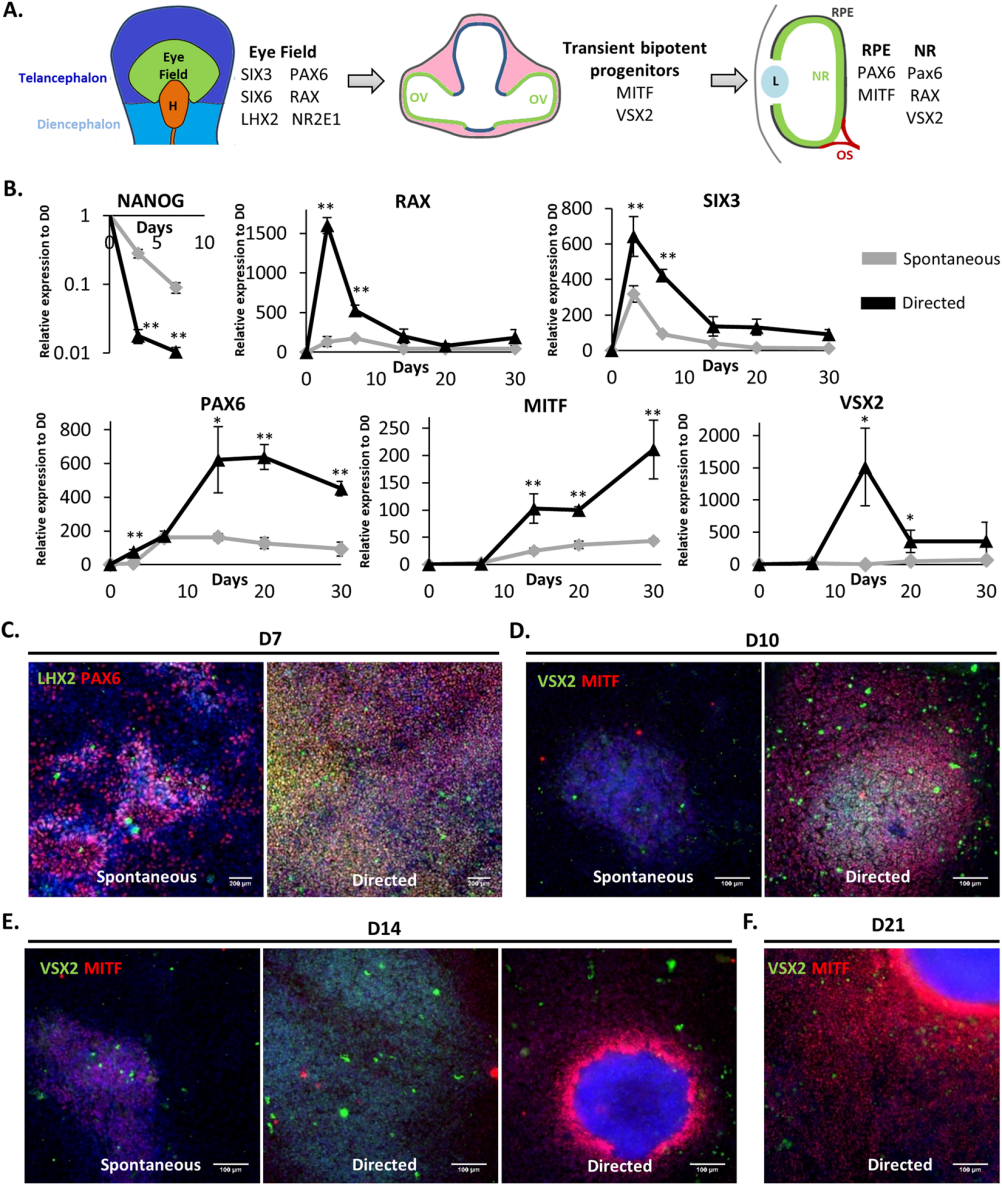
Use of nicotinamide, Activin A and ChiR99021 in a sequential manner recapitulates the main steps of retinal development. (**A**) Schematic representation of the retinal development. H, Hypothalamus; OV, Optic Vesicle; L, Lens; NR, Neural Retina; RPE, Retinal Pigment Epithelium; OS, Optic Stalk. (**B**) Relative gene expressions were quantified by RT-qPCR and normalized to mRNA expression at day 0 (n = 3, mean ± SD). Control condition corresponds to RPE 20% KSR medium. (**C**) Representative immunofluorescence for PAX6 and LHX2 at day 7 and for VSX2 and MITF at day 10 (**D**), D14 (**E**) and D21 (**F**). Nuclei are stained with DAPI (blue).

We first checked whether the sequential use of NIC, Activin A and Chir99021 could recapitulate the main steps of retinal development by evaluating the expression of markers of the early eye field stage, optic vesicle stage and immature RPE cells at different time points during the differentiation (Fig. 1A). The use of NIC for the first 7 days of differentiation significantly enhanced the transient expression of the early eye field transcription factors SIX homeobox 3 (*SIX3*) and Retinal homeobox *(RAX)* concomitantly to a higher decrease of the expression of the pluripotency marker *NANOG* at mRNA level when compared to the spontaneous protocol (p < 0.01; Fig. 1B). This eye field specification was confirmed at the protein level with the co-expression of the LIM homeobox 2 (LHX2) and the Paired box 6 (PAX6) proteins by most cells at day 7 after NIC treatment (86.8% ± 4.3%, n = 3), while only 44.3% (±2.2%, n = 3) of the non-treated cells express these two markers. Overall, our data suggested that the addition of NIC for 7 days promotes the exit of hESCs from their pluripotent state toward the eye field lineage with a better efficiency than the spontaneous differentiation.

Consecutive treatment with Activin A from day 7 to day 14 significantly increased the expression at mRNA levels of two transcription factors involved in optic vesicle patterning, the visual system homeobox 2 gene *(VSX2, also named CHX10)* and the melanocyte inducing transcription factor *(MITF)*, when compared to the spontaneous differentiation (Fig. 1B, p ≤ 0.05), with an expression peak at day 14 for *VSX2*. Concomitantly, both *RAX* and *SIX3* mRNA levels were found decreased. Induction of the optic vesicle markers VSX2 and MITF was confirmed by immunofluorescence assays. Cell clusters co-expressing these two proteins were observed by day 10 (Fig. 1D). By contrast on day 14, cells expressing VSX2 were distinct from those expressing MITF, suggesting rapid co-repression of these two genes as described previously (Fig. 1E)^33,34^.

Finally, activation of the canonical WNT signaling pathway by CHIR99021 treatment from day 14 to day 35–42 induced RPE commitment as seen by the acute decreased expression of *VSX2* mRNA levels (Fig. 1B) and the continuous increased expression of *MITF*. *MITF* expression is significantly upregulated between day 14 and day 30 in the directed protocol when compared to the spontaneous one (p < 0.01). Immunostaining assays confirmed the absence of VSX2 positive cells at day 21 and the increased number of MITF^+^ cells (87.5% ± 12.5%) (Fig. 1F). At this stage putative RPE precursors MITF-positive cells emerged and organized around 3D structures that did not express MITF and VSX2 (Fig. 1F).

**Figure 2:**
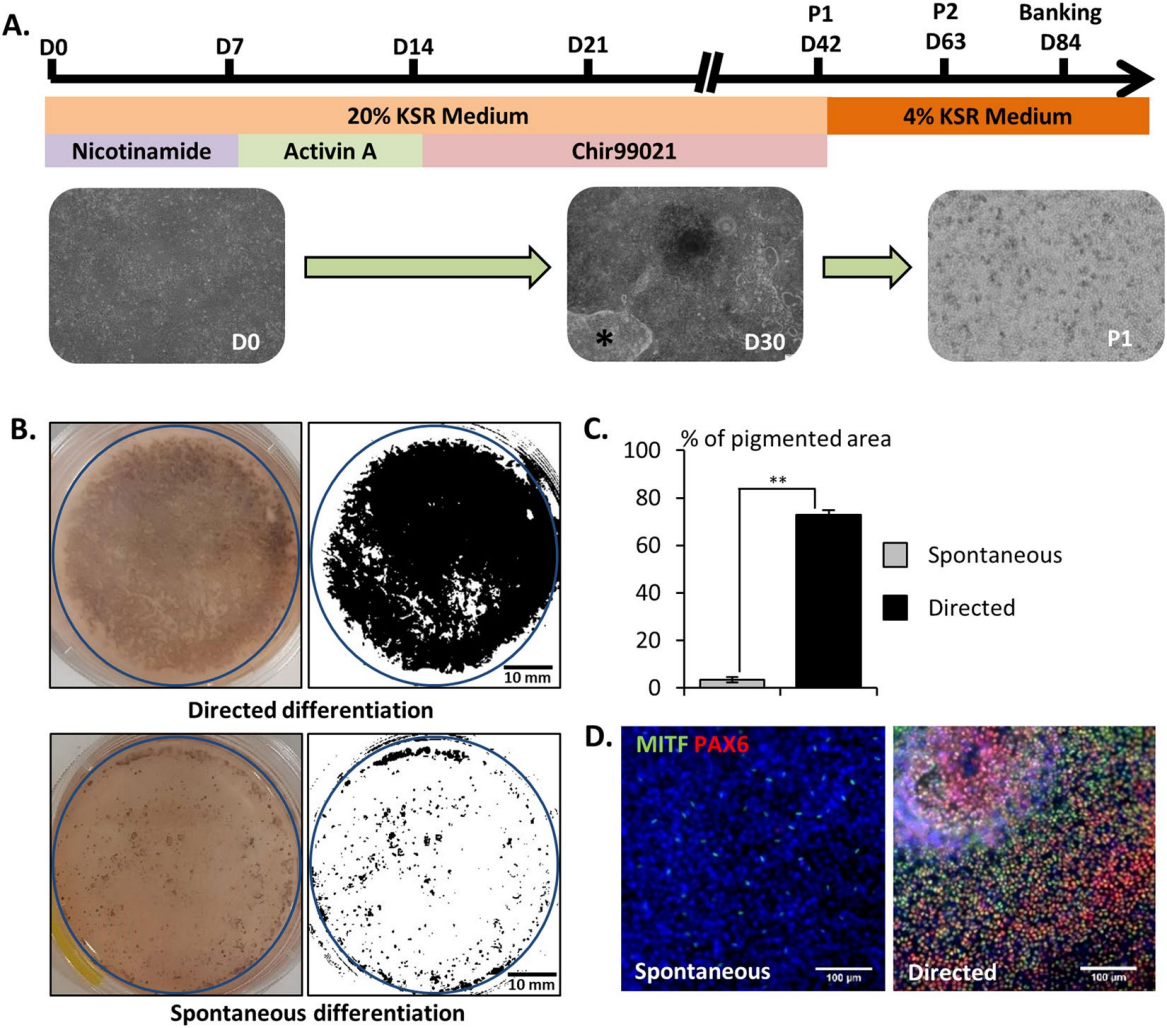
Directed differentiation protocol improves RPE differentiation. (**A**) Schematic representation of the directed differentiation protocol (black star: cell contaminants). (**B**) Representative macroscopic observation of the culture dishes after 42 days of differentiation (blue circles: quantified areas) and (**C**) quantification of the pigmented area (n = 3, mean ± SD), (**D**) Representative immunofluorescence images for the RPE markers PAX6 and MITF after 42 days of differentiation. Nuclei are stained with DAPI (blue).

We then determined the efficiency of RPE cell induction after 6 weeks of differentiation. A large majority of the culture dish with cells exposed to the directed protocol (72.96% ± 1.94% of the culture area, n = 3) was covered by pigmented cells on day 42 (Fig. 2B,C). By contrast, only isolated patches of pigmentation were visible with the spontaneous protocol (3.481% ± 1.12% of the growth area, p < 0.01) as reported in a previous study^11^ (Fig. 2B,C). Importantly, the vast majority of cells obtained after 42 days of differentiation with the directed protocol co-expressed PAX6 and MITF (82.2% ± 3.2%, n = 3), two markers of RPE cells (Fig. 2D).

Taken together our results indicate that the sequential use of Nicotinamide, Activin A and Chir99021 recapitulates the main steps of retinal development and efficiently directs the differentiation of hPSCs into a highly-enriched RPE population within 42 days compared to the spontaneous differentiation. Thus, cell differentiated through the directed protocol could be amplified directly while a prior manual selection of RPE clusters is required for the spontaneous protocol.

### The directed protocol allows obtaining a pure population of hESC-RPE cells without manual enrichment and is amenable to automation of the differentiation

**Figure 3:**
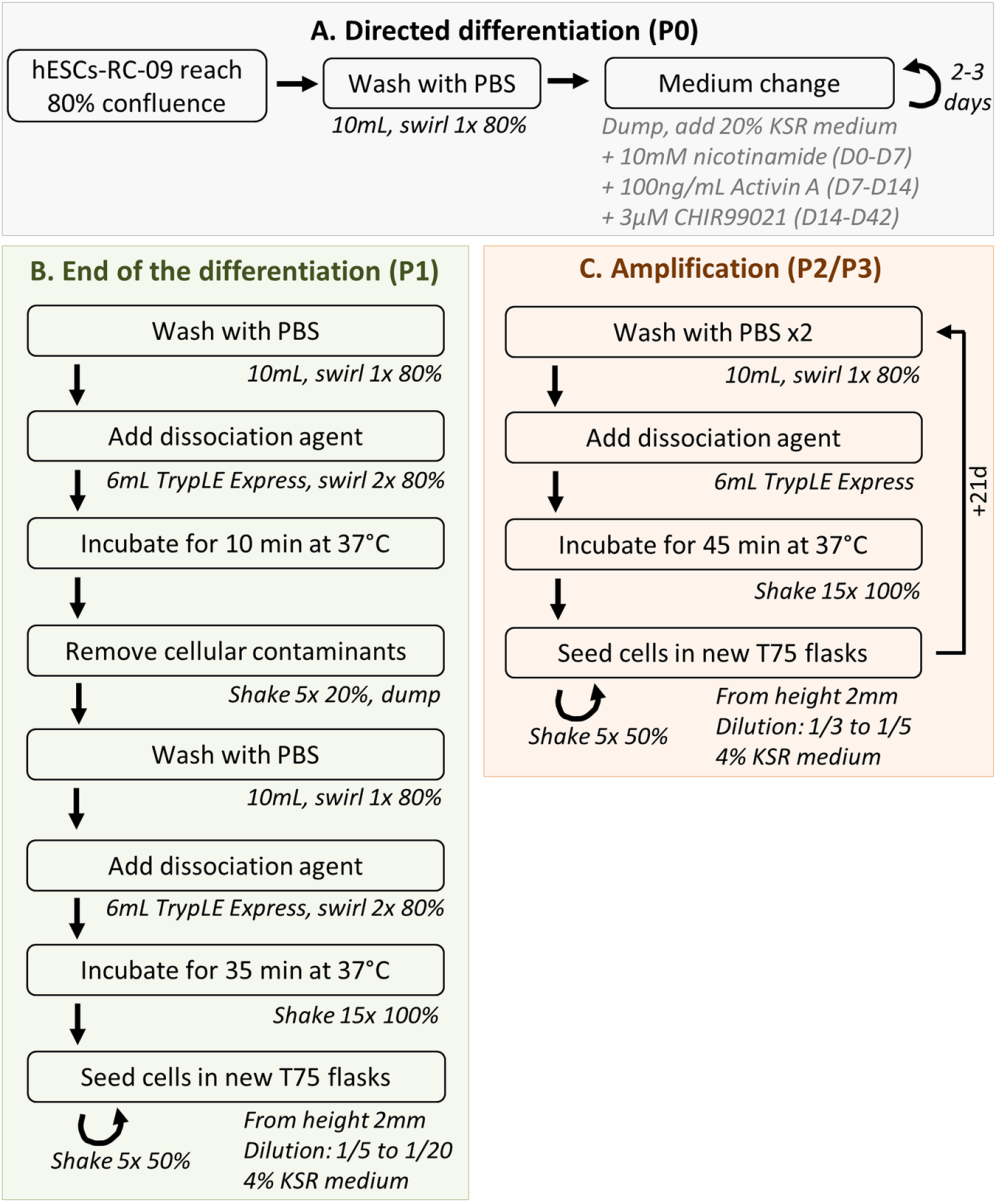
Flowchart of the automated passaging of hESC-RPE cells using the Compact Select automation platform.

Using the “directed differentiation” protocol, we set up a fully automated process by performing media changes and enzymatic passaging using the CompacT SelecT automation platform. This automated cell culture platform is composed of an incubator, bar-coded flasks for cell process tracking, multiple connected pumps to dispense culture media, a six-axis anthropomorphic robotic arm and a live-cell imaging system (Incucyte) (Figs 3 and S2).

Automation starts from the seeding of hPSCs onto 75 cm^2^ flasks. Then, cell proliferation and differentiation initiation by medium switching were performed in the robot until day 42. At this stage, hESC-RPE cells form a cohesive epithelium in culture that requires long incubation times with dissociation reagents to trigger cell detachment for further replating and amplification. In order to eliminate a maximum of cell contaminants we took advantage of this characteristic by performing a differential dissociation treatment with TrypLE Express (Fig. 3). We were able to remove the vast majority of unpigmented cells that have lower adherence to the flask than RPE cells on day 42 by applying a first short incubation of 10 minutes with TrypLE Express followed by a rinse. A second enzyme incubation of 35 minutes then enabled the detachment and dissociation of RPE cells.

As the automated system has no centrifugation capability, it was not possible to eliminate the TrypLE Express used to dissociate RPE cells. Thus, we assessed if its dilution in fresh medium after passaging did not affect the re-adherence and growth of the cells. hESC-RPE cells replated with no centrifugation step to remove the dissociation reagent after passaging at day 42 retain their proliferative capacity to form a cohesive epithelium in culture and presented no particular cell death or abnormal morphology (Fig. S3A,B). We also checked by RT-qPCR that the presence of diluted TrypLE Express did not affect RPE identity and once again no difference was detected in RPE gene expression between enzymatic passaging with or without centrifugation (Fig. S3C).

**Figure 4:**
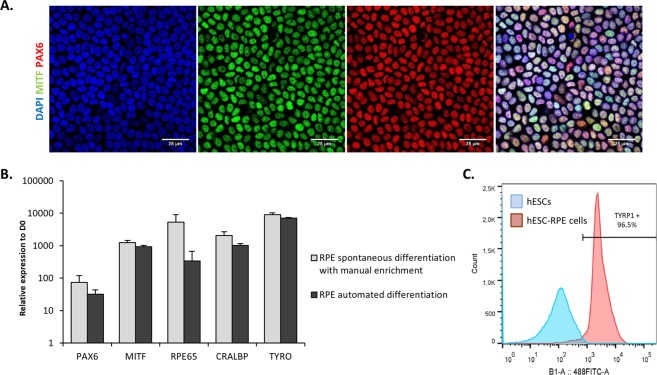
Automated differentiation and amplification of a pure population of hESC-RPE cells without manual selection. (**A**) Representative immunofluorescence and quantification for the RPE markers MITF and PAX6 at passage 2 after 21 days of culture. Nuclei are stained with DAPI. (**B**) Relative gene expression of RPE markers quantified by RT-qPCR (n = 3, mean ± SD). (**C**) Representative flow cytometry histogram for the pigmentation marker TYRP1.

After 2 automatized passages, 94.7% ± 0.2% (n = 3) of cells co-expressed the two transcription factors PAX6 and MITF (Fig. 4A) indicating a homogenous population of hESC-RPE cells comparable with the one obtained after manual enrichment^13^. The gene expression of late RPE markers such as RPE65 and CRALBP was also detected by RTqPCR at a level similar to the cells obtained with the manual spontaneous differentiation protocol (Fig. 4B). We further characterized the cell population by flow cytometry and found that 96.8% ± 1.9 (n = 3) of the cells expressed the pigmentation marker tyrosinase related protein 1 (TYRP1) at passage 2 (Fig. 4C).

All together these data demonstrate that we were able to obtain pure *bona fide* hESC-RPE cells in an automated system with a quality similar to the cells obtained through the widely used spontaneous differentiation method.

### hESC-RPE cells obtained by an automated differentiation are mature and functional

**Figure 5:**
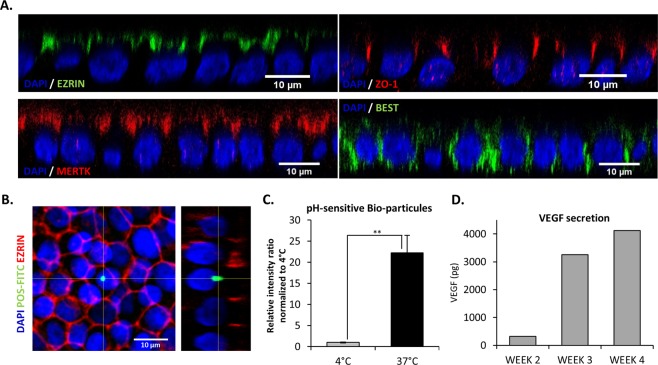
hESC-RPE cells obtained by automated differentiation are mature and functional. (**A**) Confocal images (projection of XZ planes) for the RPE markers EZRIN, BEST, ZO-1 and MERTK. Nuclei are stained with DAPI. (**B**) Confocal Images (projection of YZ planes) of hESC-RPE cells after 24 hours of exposure to fluorescein isothiocyanate (FITC)–labeled pig photoreceptor cell outer segments (FITC-POS, green; EZRIN, red; DAPI, blue). (**C**) Fluorescence intensity quantification of hESC-RPE cells after exposure to pH-sensitive bioparticles (n = 3, mean ± SD). (**D**) Quantification of vascular endothelial growth factor (VEGF) secreted by hESC-RPE cells at different time points using an ELISA assay (Representative assay).

Important issues concerning cells differentiated from hPSCs are their maturity and functionality. As an indicator of epithelial maturity, we evaluated the apico-basal polarization of specific RPE markers. As expected, hESC-RPE cells homogeneously expressed the microvilli protein EZRIN (95.0% ± 2.8%, n = 3), the tight junction marker Zonula Occludens-1 (ZO-1, 99.3% ± 0.4%, n = 3) and the MER proto-oncogene tyrosine kinase receptor (MERTK, 97.1% ± 1.1%, n = 3) at their apical membrane while the calcium activated chloride channel, BESTROPHIN (BEST, 89.4% ± 3.9%, n = 3) was localized at the baso-lateral compartment (Figs 5A and S4).

One of the most important functions of RPE cells is the phagocytosis of the outer segments shed by the photoreceptors. To determine whether the cells differentiated according to our directed protocol on the automated cell culture platform were functional, we assessed their ability to phagocyte pig fluorescein isothiocyanate (FITC)–labeled photoreceptor outer segments and quantified the fluorescence signal of pH-sensitive particles that become fluorescent after cell entry and phagosome formation. hESC-RPE cells were able to phagocyte FITC-labeled photoreceptor outer segments as shown by the cytoplasmic localization of the FITC signal under the apical limit Ezrin positive (Fig. 5B). hESC-RPE cells incubated with pH-sensitive particles at 37 °C had a fluorescence intensity 22.2 fold higher compared to cells incubated at 4 °C, a temperature that inhibits the phagocytic process (Fig. 5C). Another indicator of RPE functionality is the ability to secrete a wide range of growth factors including the vascular endothelial growth factor (VEGF). We quantified the secretion of VEGF after several culture weeks and we observed a progressive increased of VEGF secretion starting from 2 weeks of culture (Fig. 5D).

All these results indicate that RPE cells differentiated from hPSCs using a fully automated protocol are functional *in vitro*.

### hESC-RPE cells differentiated through automation can be amplified until passage 3 to produce large cell banks

**Figure 6:**
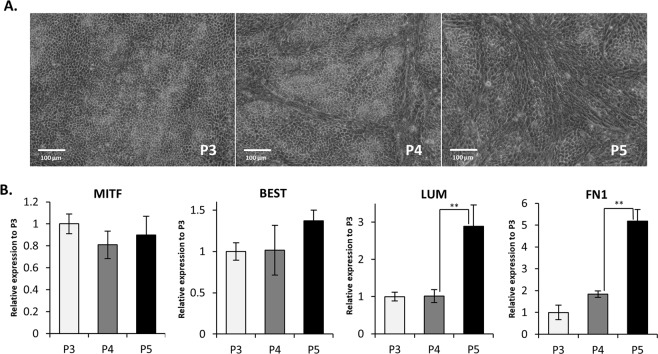
hESC-RPE cells obtained by automated differentiation can be maintained in culture until passage 3 before starting an EMT. (**A**) Light microscopy images of hESC-RPE cells at passage 3, 4 and 5 at day 21. (**B**) Relative gene expression of EMT (LUM and FN1) and RPE (MITF and BEST) markers quantified by RT-qPCR (n = 3, mean ± SD).

Previous studies showed that hESC-RPE cells had a limited amplification potential before they undergo an epithelial-mesenchymal transition (EMT)^35,36^. In line with these studies, hESC-RPE cells obtained with an automated process adopted a mesenchymal phenotype starting from passage 4 (Fig. 6A) despite the maintenance of the gene expression of the RPE markers *MITF* and *BEST* (Fig. 6B). Indeed, the cells switched from a classical cobblestone organization to elongated cell morphology (Fig. 6A). This microscopic observation is correlated with the rising expression of mesenchymal markers LUMICAN (*LUM*) and FIBRONECTIN 1 (*FN1*), two extracellular matrix proteins, starting at passage 5 when compared to passage 3 and 4 (p < 0.01; Fig. 6B). This suggests an EMT transition of hESC-RPE cells, which however maintain an RPE identity. Consequently, we decided to bank our cells at passage 2 using an automated cell banking system (Fill-it, Sartorius) to obtain *bona fide* hESC-RPE cells at passage 3 after thawing.

## Discussion

Since the publication of the first protocol showing that hESCs spontaneously differentiate into RPE cells, extensive efforts have been made to reduce the duration and increase the efficiency of differentiation protocols^17,18,20,24^. Previous works described that the sequential use of NIC and Activin A allowed increasing embryonic body commitment into retinal lineage. In another hand, *Leach et al*. showed that the same factors, in addition to many others including bFGF, Noggin, DKK1 (Dickkopf WNT signaling pathway inhibitor 1), Insulin Growth Factor (IGF)-1, Chir99021, B27 and N2 supplements, increased RPE cell differentiation of hPSCs^21^. We demonstrated in this study that most of these cytokines and supplements were not essential to trigger an efficient and pure RPE cell differentiation. Indeed, the use of only 3 compounds (NIC, Activin A and CHIR99021) in a sequential manner allowed obtaining a pure population of RPE cells without 3-dimensional culture and manual dissection of pigmented foci during the differentiation process. This optimized differentiation is thus amenable to automation.

As previously described^20,24^, the use of Nicotinamide quickly reduced the expression of pluripotent markers, suggesting the rapid exit from pluripotent state, while increasing eye field genes expression in treated cells. In this study, Activin A increased both expressions of the neural retina marker *VSX2* and the RPE marker *MITF*. These two markers are initially co-expressed by the same cells at day 10 suggesting that at this stage the cells could be bipotent^37^ and able to give rise to both RPE and neural retina progenitors. But quickly, two subpopulations of cells expressing either one of these two genes appeared. This result is consistent with the known role of *VSX2* on *MITF* repression during optic vesicle patterning^33^. The increased *VSX2* expression following Activin A treatment is however surprising as it is known to induce RPE specification rather than neural retina^38^. This result could be due to the endogenous expression of bFGF that positively regulates the expression of *VSX2*. The simultaneous use of a FGF inhibitor, such as SU5402, already used in a previously described protocol, may be necessary at this stage to repress neural retina markers^39^. The presence of cells expressing only *VSX2*, meaning that they are potentially engaged into the neural retinal lineage, could be a concern at this stage. However, the later use of the small molecule CHIR99021 rapidly leads to the acute decrease of VSX2 expression concomitantly with an increased expression of *MITF*, indicating that the activation of the Wnt/β-catenin pathway at this stage might induce the death of *VSX2* positive cells or their conversion into RPE precursors. CHIR99021 treatment could also induce the differentiation of VSX2 positive cells into other mature lineages that are further eliminated by the differential dissociation.

The “directed protocol” developed in this study enabled us to obtain 87.5% of RPE precursors that expressed MITF by day 21. However, cells were maintained further in culture in order to allow more maturation. Once a cohesive epithelium is formed at day 42, cells were passaged. This cohesive epithelium is suitable to a differential dissociation that eliminates the last cell contaminants without manual enrichment. Compared with the protocol published by *Foltz et al*.^22^ where cells are banked at day 84, we bank hESC-RPE cells with the same timing (D84). Thus, the removal of factors other than NIC, Activin A and CHIR99021, used in Foltz and collaborators study did not delay the overall production of hPSC-RPE cells nor affected the quality of the cells obtained.

Using the automated differentiation process described in this study, it is theoretically possible to produce about 16 billion of hESC-RPE cells at passage 2 per batch (Fig. S5). A bank of this size is much larger than those previously described by us and others^13,17,40^ that range from 0.05 to 0.8 × 10^9^ cells, and could be produced by a single operator supervising the robot. Moreover, the use of HYPERflask (Corning) with a growth area of 1720 cm^2^ (compared to 75 cm^2^ flask used in this study) could even dramatically increase the number of cells produced per batch. Another way to increase the size of the bank would be to delay the EMT. Indeed, the number of passages without EMT might be extended as previously described by the addition of a ROCK inhibitor in the culture medium^35^.

hPSC-RPE cells have been already grafted in AMD patients either as a cell suspension^15,41,42^ or a polarized epithelium resting on a synthetic basement membrane^14,43^. The use of cell suspension formulation considerably simplifies the logistical and surgical procedures but several studies made in animal models, including our own, suggest that the survival of the RPE cells and the visual benefits for the animal are improved when the cells are grafted as an epithelial tissue rather than a cell suspension^13,44^. In human, these two approaches have shown both satisfactory safety results and promising efficacy results, even if the extent and the causes of visual improvement in transplant recipients remain ambiguous^16^. Nevertheless, considering that 1 × 10^5^ hESC-RPE cells are currently used to graft a human eye with the both methods^14,15,43^, the automated process presented here should allow to produce enough cells to treat several thousands of patients with retinal degeneration even if some steps of the production, such as the simultaneous banking of a huge numbers of cryovials, remains challenging. The automated culture system could be qualified for clinical cell productions, as actually already done by others^45^. However, before reaching a clinical application, the raw materials used with this automated protocol might be adjusted to comply with Good Manufacturing Practice (GMP) guidelines.

In conclusion, following the previously published amplification of hPSCs using CompacT SelecT automate^46^, we described a fully automated RPE cell differentiation process from the hPSCs thawing to the banking of differentiated cells. Such automated process is a step towards the scale up and the industrialization of RPE differentiation that will be necessary to treat large numbers of patients. Finally, any differentiation protocol that doesn’t require 3D culture or manual selection could theoretically be adapted to this automated culture system opening new perspectives concerning the scale up and the industrialization of the production of many cell types differentiated from hPSCs.

## Materials and Methods

### Manual hESCs culture and RPE cell differentiation

Clinical-grade hESC line RC-09^13,47^ was used and cultured in feeder free conditions using mTeSR™1 Medium (StemCell technologies) and hESC qualified Matrigel (Corning) (Fig. S1). Cells were banked at passage 36 and used for RPE differentiation between passage 38 and 45. Cells were plated at 5 × 10^4^ cells per cm^2^ and grown until they reached 80 percent of confluence before switching to a differentiation medium composed of Dulbecco’s modified Eagle’s medium (high glucose, Thermo Fisher Scientific) supplemented with 50 μM β-mercaptoethanol, 1× minimum essential medium–nonessential amino acids (Thermo Fisher Scientific) and 20% (D0-D42) or 4% (after passage 1) of knockout serum replacement (KSR, Thermo Fisher Scientific). During all the differentiation process the medium was changed every 2/3 days.

hESC-RPE cells were obtained by spontaneous differentiation of hESCs as previously described^25^. Briefly, hESCs were grown to confluence and switched to a bFGF deprived culture medium. Pigmented patches were then dissected under a stereomicroscope with a fine 15° ophthalmic knife and plated onto culture dishes coated with hESC qualified Matrigel (corning).

For the referred “directed differentiation” protocol, 10 mM Nicotinamide (Sigma), 100 ng/ml Activin A (Peprotech) and 3 µM CHIR99021 (Tocris) were added sequentially to the basal differentiation medium at specific time points (Fig. 2A). On day 42, cells were incubated with TrypLE Express Reagent (Thermo Fisher Scientific) for 10 minutes to remove cell contaminants, then washed with PBS and re-incubated with TrypLE Express Reagent for 35 minutes to allow RPE dissociation. Cells were then seeded at a final dilution of 1/5 in dishes coated with hESC qualified Matrigel (Corning).

Mature hESC-RPE cells were dissociated and cryopreserved in liquid nitrogen vapors with CryoStor CS10 medium (StemCell technologies) at passage 1 or 2.

### Automated RPE differentiation process

The CompacT SelecT (Sartorius) is a fully automated cell culture platform which allows the expansion and differentiation of large batches of adherent cells in a controlled environment (Fig. S2). The system allows the automation of media changes and cell passaging as well as the monitoring of culture vessels with the automated live-cell imaging system IncuCyte (Sartorius). Contrary to the manual protocol, cells were not centrifuged after dissociation but were directly seeded into new flasks with enough medium to ensure that the final concentration of TrypLE Express reagent in the daughter flasks did not exceed 5%. The automated process is presented in Fig. 3.

Automated medium changes consisted in (i) retrieving T-flask from the incubator, removing flask cap and pouring media into a waste funnel, (ii) adding the appropriate culture medium using the peristaltic pump system (“dispensing” step), and (iii) replacing T-flask in the flask carousel incubator. Automated passaging consisted in step (i) described above and (ii) add PBS, (iii) swirl flask with the robot arm to ensure an even coverage of PBS across the cell sheet to wash cells, (iv) empty flask, (v) add TrypLE Express, swirl flask and incubate at 37 °C for 45 min, (vi) add fresh medium to dilute the dissociation reagent, (vii) shake flask from side-to-side at a given speed a number of times to dissociate the cell sheet into single cells or small clumps (“shaking” step), (vii) seed the cell suspension into new T-flasks with a split ratio of 1:3 or 1:5, swirl and replace in the incubator (“seeding” step). Step (v) was modified for the first cell passaging at the end of the differentiation (P0) to perform a two-step enzymatic dissociation procedure to enrich the culture for RPE cells by: (i) incubating the cells for 10 min with TrypLE Express, (ii) gently shaking the flask and pouring the dissociation agent into a waste funnel to remove non-RPE cells, (iii) incubating the cells for 35 min with TrypLE Express (“differential dissociation” step).

### Quantitative real-time polymerase chain reaction

Total RNAs were extracted using RNAeasy Plus Mini kit (Qiagen) and cDNA synthesized using SuperScript III (Invitrogen). Quantitative real-time RT-PCR was performed using a Quant Studio 12 K flex (Applied Biosystems) with HiGreen qPCR Master Mix (Thermo Fisher Scientific). Primer sequences are listed in Supplementary Table 1. Experiments were performed with at least three technical replicates per plate and expression levels were normalized to 18S. Relative expression compared to hESCs gene expression levels were determined by calculating the 2^−ΔΔCt^.

### Immunostaining

hESC-RPE cells were grown on Matrigel-coated 96 or 24-well plates. Adherent cells were fixed in 4% PFA for 10 min at room temperature (RT) and rinsed 3 times with PBS. After 30 min in blocking solution (10% FBS in 0.1% Triton PBS) at RT, cells were incubated with primary antibodies overnight at 4 °C (Antibodies are listed in Supplementary Table 2). After 3 washes in PBS, appropriate Alexa Fluor-conjugated secondary antibodies (Invitrogen) were added at 1:500 for 1 h at RT in presence of DAPI (Invitrogen).

### Image acquisition and analysis

Images were acquired with an Axio observer Z1 microscope (Zeiss) with a Hamamatsu ORCA-flash 4.0 camera and a spinning disk unit (Yokogawa CSU-X1-A1N-E; Camera evolve, EMCCD 512) with Metamorph software or with a LSM-800 confocal microscope (Zeiss) with Zen software. Images were exported, analyzed and processed with Fiji software. For zx images, xy stacks (0.33 μm z step size) covering cell width were resliced in zx. The quantification of pigmented areas was performed after manual delimitation of culture dish areas using Fiji software. Pictures were then binarized to 8-bit images using a fixed intensity threshold and the black area fraction was measured.

### Flow cytometry

Cells were detached from culture plates, fixed in 4% PFA for 10 min at RT and permeabilized with PBS containing 0.1% Triton for 30 min before labeling with TYRP1 antibody for 1 hr at RT. Labeling of the cell surface markers TRA-1-81 and SSEA4 was performed on freshly dissociated cells for 15 min at 4 °C. Cells were then incubated with fluorochrome-conjugated primary antibody for 30 min at RT and rinsed twice with PBS. The antibodies used and their working dilutions are listed in Supplementary Table 2. Cells were analyzed using a cell MACSquant analyzer (MiltenyiBiotec). Gates were drawn according to fluorescence minus one (FMO) controls or on samples labeled with isotype control antibodies. Data were analyzed using FlowJo software (Tree Star, Ashland, OR).

### Phagocytosis assay

hESC-RPE cells were exposed for 24 hours to purified FITC-labeled photoreceptor outer segments of pig (gift from Dr. E. Nandrot). After washing with PBS, cells were fixed in cold methanol and labelled with DAPI. Images were taken with LSM-800 confocal microscope (Zeiss). hESCs derived RPE cells were also exposed to pHrodo Green Zymosan Bioparticles (Thermo Fisher Scientific) overnight at 37 °C. These particles are pH-sensitive and become fluorescent after cell entry and phagosome formation. As a negative control, phagocytosis assays were performed at 4 °C to block the phagocytic process. Plates were then read using a microplate reader (Clariostar-BMG LABTECH) and values were normalized to DAPI intensities.

### VEGF quantification by ELISA assay

VEGF measurements were done in triplicate using the human VEGF Quantikine ELISA kit (R&D System) according to manufacturer instruction.

### Statistical analysis

All experiments were performed in triplicate. Summary statistical analyses were performed in XLSTAT software. Comparisons between experiments were performed using the unpaired t-test and statistical significance was established as *p < 0.05, **p < 0.01.

## Supplementary information

Supplementary informations

## Data Availability

Additional data and information for reproducing the results described in the article are available from the corresponding author on reasonable request.
