# Supplementary material for: Automation of human pluripotent stem cell differentiation toward retinal pigment epithelial cells for large-scale productions
Source: Sci Rep. 2019 Jul 23;9:10646. doi: 10.1038/s41598-019-47123-6 (PMC6650487; doi:10.1038/s41598-019-47123-6)
Supplement: Supplementary file 1 — Supplementary informations [file 41598_2019_47123_MOESM1_ESM.pdf]

Automation of human pluripotent stem cell differentiation toward retinal pigment epithelial cells for large-scale productions

Florian Regent[1,2], Lise Morizur[1,2,3], Léa Lesueur[1,2,3], Walter Habeler[1,2,3], Alexandra Plancheron[1,2,3], Karim Ben M'barek[1,2,3], Christelle Monville[1,2*]

[1]INSERM U861, I-Stem, AFM, Institute for Stem cell Therapy and Exploration of Monogenic diseases, 91100 Corbeil-Essonnes, France;

[2]UEVE U861, I-Stem, AFM, Institute for Stem cell Therapy and Exploration of Monogenic diseases, 91100 Corbeil-Essonnes, France;

[3]CECS, I-Stem, AFM, Institute for Stem cell Therapy and Exploration of Monogenic diseases, 91100 Corbeil-Essonnes, France;

*cmonville@istem.fr

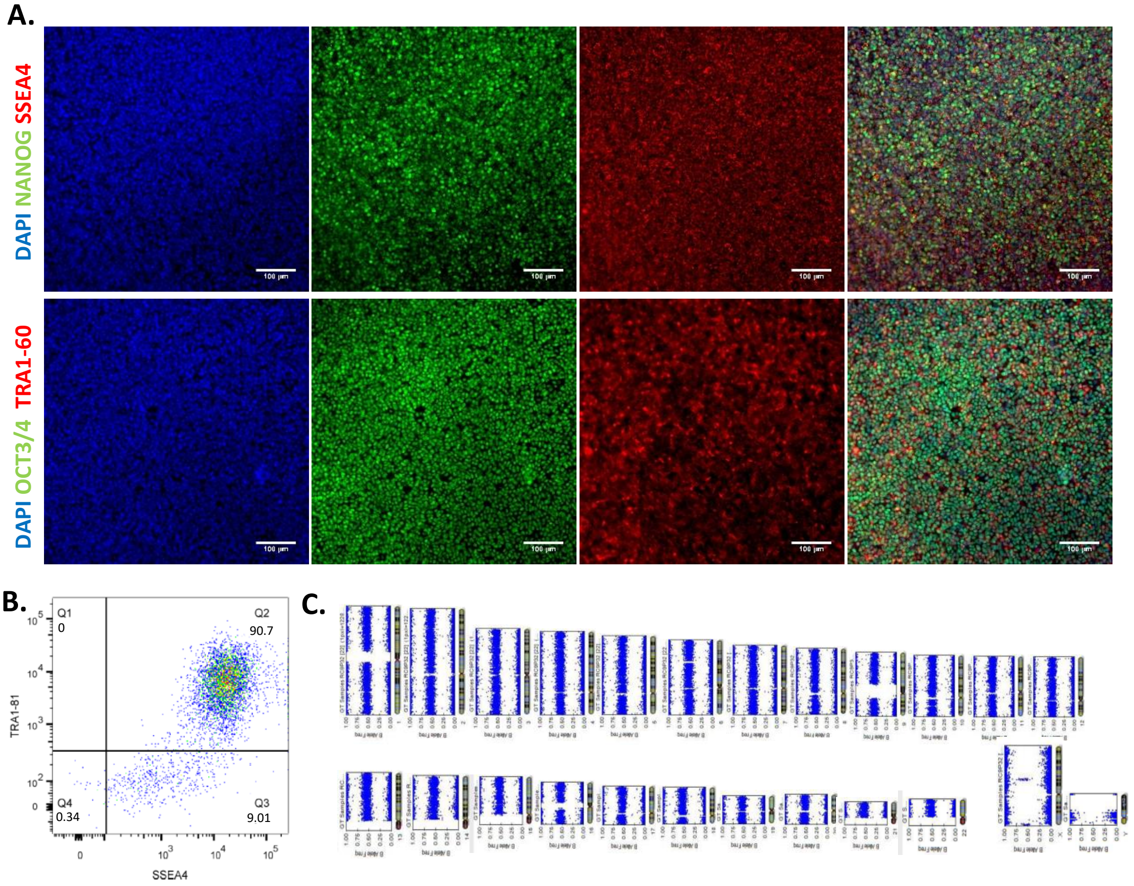

Figure S1 : Characterization of the clinical-grade hESC line RC-09. (A) Fluorescence images for the pluripotent markers NANOG, SSEA4, OCT3/4 and TRA1-60. Nuclei are stained with DAPI. (B) Representative flow cytometry histogram for the pluripotency markers TRA1-81 and SSEA4. (C) Analysis of copy number chromosome using high-density single-nucleotide polymorphism-based genotype arrays.

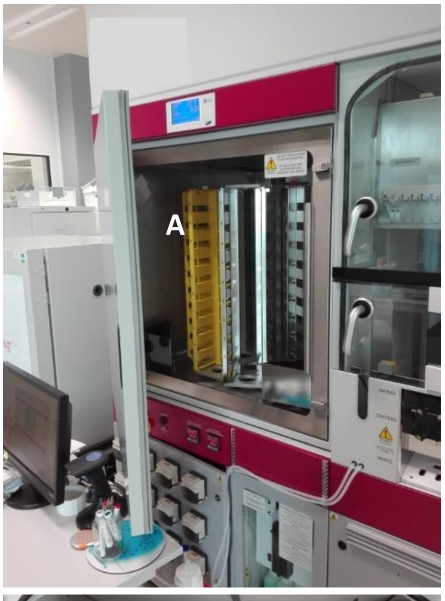

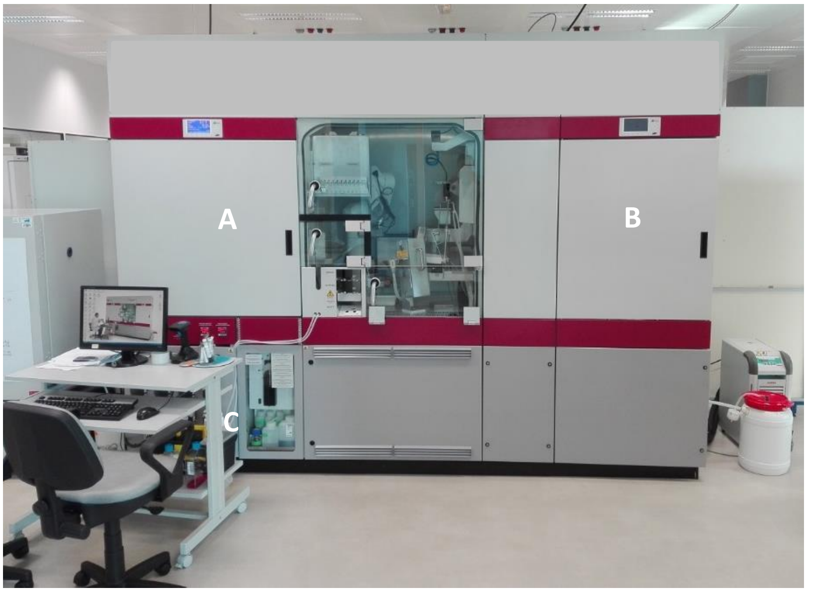

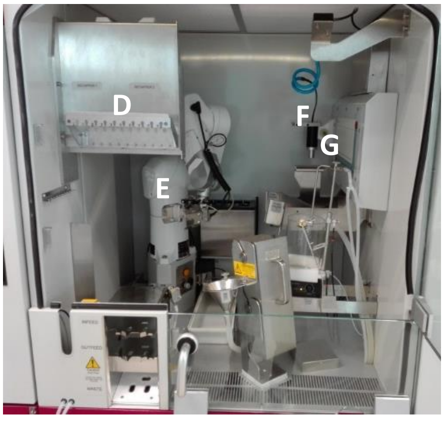

Figure S2 : Overview of the CompacT SelecT platform. (A) Flask carousel incubator, (B) Plate incubator, (C) Media pumps, (D) Decappers, (E) Robot arm, (F) Pipette head and (G) IncuCyte live-cell analysis System .

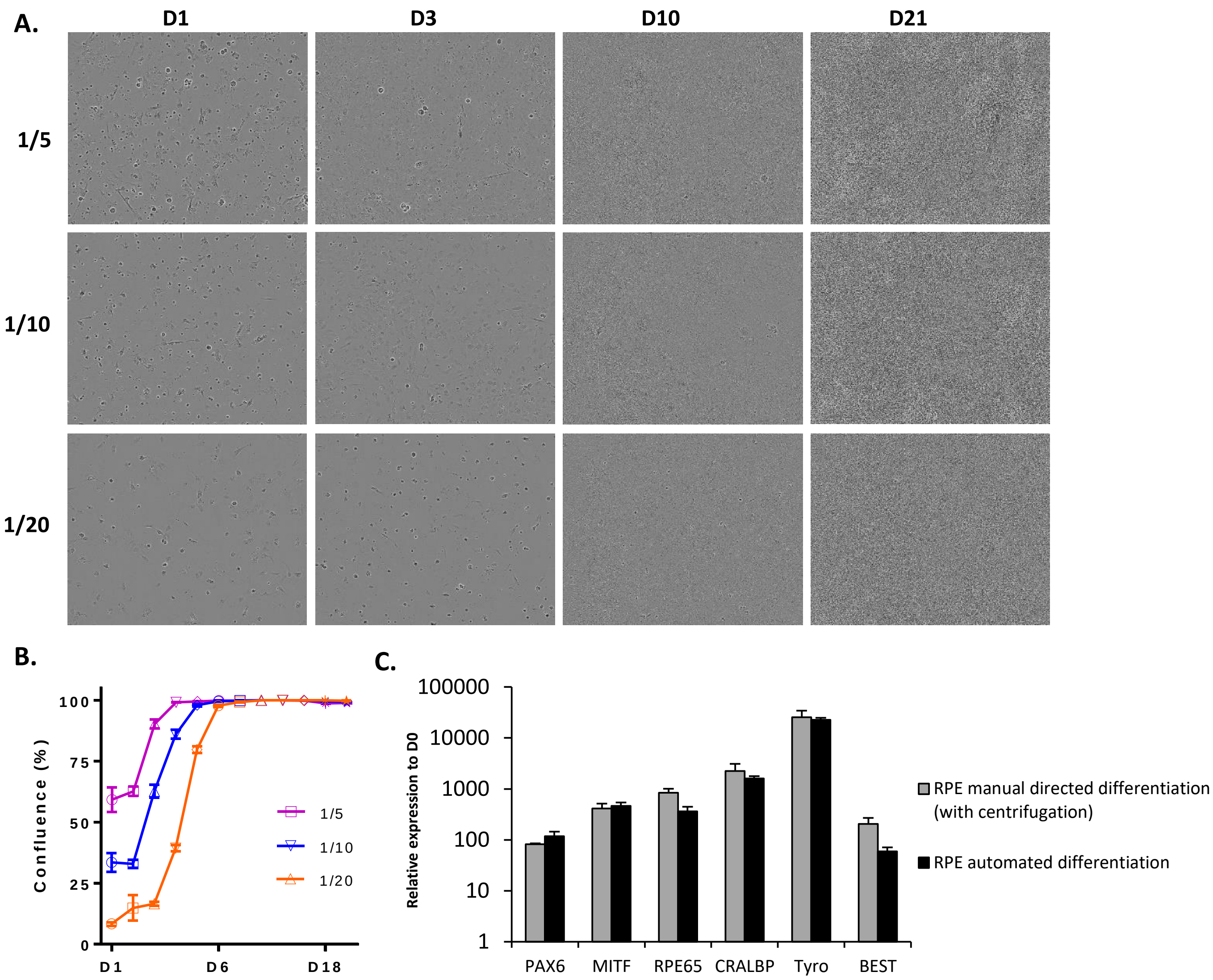

Figure S3. Automated passaging of hESC-RPE cells with the absence of a centrifugation step to remove the dissociation reagent does not affect their identity. Light microscopy images (A) and quantification of the cell confluence (B) of hESC-RPE cells split by 5, 10 or 20 after 42 days of differentiation without centrifugation after dissociation using the CompacT SelecT platform. (C) Relative gene expression of RPE markers quantified by RT-qPCR (n = 3, mean ± SD).

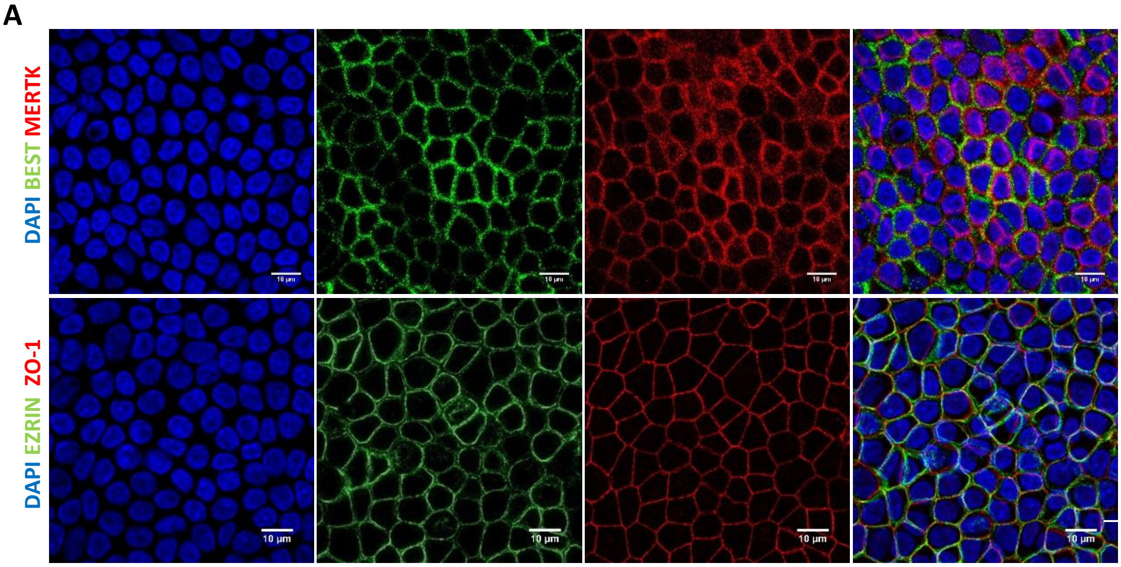

Figure S4 : Characterization of hESC-RPE cells. (A) Fluorescence images for the RPE markers BEST, MERTK, EZRIN and ZO-1 at passage 2 day 35. Nuclei are stained with DAPI.

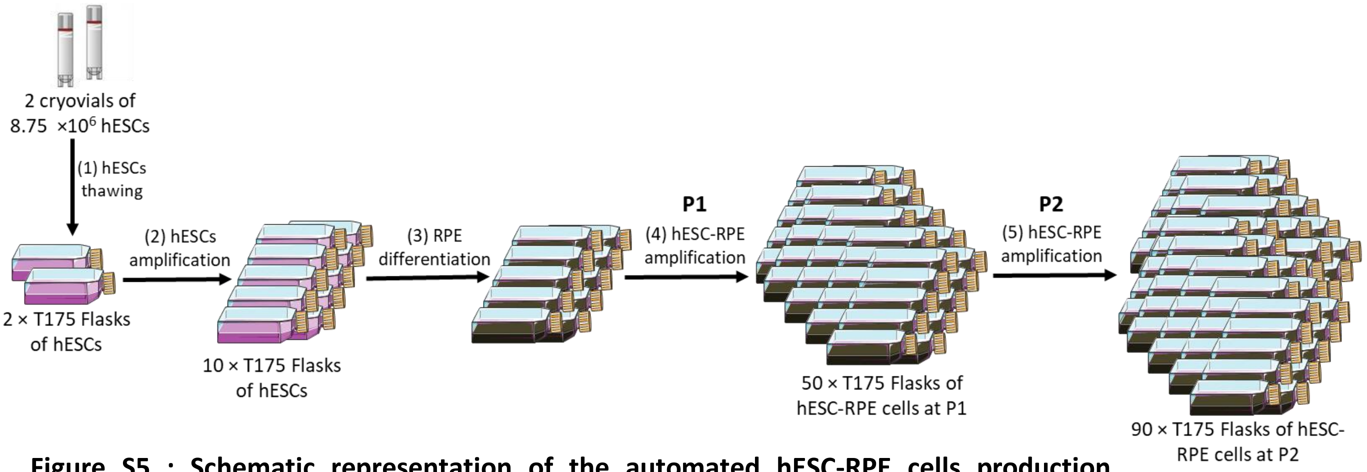

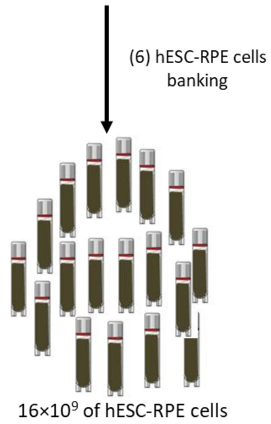

Figure S5 : Schematic representation of the automated hESC-RPE cells production process. Steps (2) to (5) can be performed using the CompacT SelecT system automation platform. Step (6) can be performed using automated cryovial filing system Fill-it and the controlled-rate freezing system Cryomed. This figure was adapted with the use of Servier Medical Art images, licence: CC B-Y 3.0.

Gene		SEQUENCE
18S	FRW	GAGGATGAGGTGGAACGTGT
	REV	TCTTCAGTCGCTCCAGGTCT
NANOG	FRW	CAAAGGCAAACAACCCACTT
	REV	TCTGCTGGAGGCTGAGGTAT
RAX	FRW	GGCAAGGTCAACCTACCAGAG
	REV	CATGGAGGACACTTCCAGCTT
SIX3	FRW	CCTCCCACTTCTTGTTGCCA
	REV	CGCTACTCGCCAGAAGTATGG
PAX6	FRW	GCCAGCAACACACCTAGTCA
	REV	TGTGAGGGCTGTGTCTGTTC
VSX2	FRW	CTGCCGGAAGACAGGATACA
	REV	TAGAGCCCATACTCCGCCA
MITF	FRW	CCGGGTGCAGAATTGTAACT
	REV	GGACAATTTTGGCATTTTGG
RPE65	FRW	AGCACTGAGTTGAGCAAGCA
	REV	GGCCTGTCTCACAGAGGAAG
CRALBP	FRW	CACGCTGCCCAAGTATGATG
	REV	CCAGGACAGTTGAGGAGAGG
TYROSYNASE	FRW	GTGTAGCCTTCTTCCAACTCAG
	REV	GTTCCTCATTACCAAATAGCATCC
BEST1	FRW	GTCAGAGGCTCCTCCTTCCT
	REV	TCTGCTCCACCAGTGTTCTG
LUM	FRW	CTTCAATCAGATAGCCAGACTGC
	REV	AGCCAGTTCGTTGTGAGATAAAC
FN1	FRW	GGAAAGTGTCCCTATCTCTGATACC
	REV	AATGTTGGTGAATCGCAGGT

Table S1 : List of quantitative reverse transcriptase polymerase chain reaction (qRT-PCR) primers.

Antibody	Host	Company	Reference	Dilution	Application
PAX6	Rabbit	Biolegend	PRB-278P	1/500	Immunofluorescence
MITF	Mouse	Dako	M3621	1/250	Immunofluorescence
VSX2	Goat	Santa Cruz Biotechnology	sc-21690	1/250	Immunofluorescence
TYRP1	Mouse	LifeSpan BioSciences	MS-771-P1	1/500 - 1/100	Flow cytometry
EZRIN	Mouse	Sigma	E8897	1/250	Immunofluorescence
ZO-1	Rabbit	Invitrogen	402300	1/500	Immunofluorescence
BEST	Mouse	Abcam	ab2182	1/250	Immunofluorescence
MERTK	Rabbit	Abcam	Y323	1/500	Immunofluorescence
NANOG	Rabbit	Abcam	ab80892	1/500	Immunofluorescence
OCT 3/4	Goat	Santa Cruz Biotechnology	sc-5279	1/500	Immunofluorescence
SSEA4	Mouse	R&D systems	FAB1435A	1/100	Flow cytometry
TRA1-60	Mouse	Santa Cruz Biotechnology	sc-21705	1/500	Immunofluorescence
TRA1-81	Mouse	Santa Cruz Biotechnology	sc-21706	1/500	Immunofluorescence
TRA1-81	Mouse	eBioscience	12-8883-82	1/100	Flow cytometry

Table S2 : List of primary antibodies.
